# Voices for gender equity in medical physics

**DOI:** 10.1002/acm2.12487

**Published:** 2018-11-08

**Authors:** Julianne M. Pollard‐Larkin, Kelly C. Paradis, Jean M. Moran, Mary K. Martel, Yi Rong

**Affiliations:** ^1^ Department of Radiation Physics The University of Texas M.D. Anderson Cancer Center Huston TX USA; ^2^ Department of Radiation Oncology University of Michigan Ann Arbor MI USA; ^3^ Department of Radiation Oncology University of California Davis Comprehensive Cancer Center Sacramento CA USA

## 1 INTRODUCTION

### 1.A Yi Rong, PhD

This past year has delivered many headlines in the media with regard to gender‐based misconduct and inequity in the workplace, in publicly funded programs, and in corporate boardrooms. In reflection on our own field of medical physics, it should be noted that our AAPM leadership has devoted substantial efforts in addressing gender disparity. Among all the fields represented by the American Institute of Physics, our field may be the most gender‐equitable field of a physics specialty in North America.^1^ Almost every job posting on the AAPM website has a declaration of employer support toward gender equity, yet the AAPM 2017 salary survey reported 23% (574/2470) female physicists compared with 77% (1896/2470) male physicists among those who have taken the survey. Sadly, rather than an improvement in equity, we may be losing ground compared to a 2013 survey reporting 25% female vs 75% male in medical physics profession.^2^ What are we missing here? Are we just using the expression “gender equity” as an obligatory disclaimer for job postings, or a must‐have slogan for a leadership position? Now let's take a self‐reflection test: If you hear a statement, “Thank goodness we have a smart and reliable resident covering our routine QA. We are so short‐handed since a colleague just quit for family reasons”, was the “smart and reliable” resident you envision in this statement male? Were you picturing a female physicist quitting her job? Questions like these can help us realize our own unconscious gender biases. These biases exist in every one of us, and ultimately affect our decisions on a wide range of workplace decisions. To truly “Promote gender equity”, it requires fundamental training in overcoming conscious and/or unconscious biases, and it appears we have a major challenge in addressing them. This JACMP editorial will present four “Parallel” voices of our female colleagues, each with their own unique backgrounds and stories.

Dr. Julianne Pollard‐Larkin recently published a piece in the ASTRO 2108 Summer Newsletter titled “Medical Physics: The Most Gender Equitable Physics Specialty”.^1^ Her article highlighted a confirmed trend of steadily increasing numbers of women trainees in Medical Physics. She is a Physics service chief in the Thoracic Service at MD Anderson Cancer Center, the chair for AAPM's Diversity and Inclusion subcommittee, and she graduated from MD Anderson's Medical Physics residency program nearly a decade ago. Over the course of her career and training, she has seen the evolution of gender equality at two major academic institutions and within AAPM and has some ideas on the best way to promote gender equality in our field.

Dr. Kelly C. Paradis (née Younge) earned her Ph.D in Physics in 2010 from the University of Michigan, studying the use of Rydberg atoms for quantum information processing. She completed her residency in clinical and academic medical physics at University of Michigan in 2013. Dr. Paradis worked as a staff medical physicist for Trillium Health Centers in Ontario, Canada, and then returned to University of Michigan where she joined the faculty in the Department of Radiation Oncology as Assistant Professor. Dr. Paradis has authored and co‐authored 29 peer‐reviewed papers, and has a strong interest in patient safety, risk analysis, peer mentoring, as well as diversity, equity, and inclusion (DEI) topics.

Dr. Jean Moran is Professor and Co‐Director of Physics at the University of Michigan, Associate Director of the Michigan Radiation Oncology Quality Consortium, and Chair of the AAPM's Therapy Physics Committee. She has been a tireless advocate for gender equity throughout her career. In 1990, she served as an undergraduate on a Provost committee on sexual harassment at the Massachusetts Institute of Technology. As a graduate student at the University of Michigan, she served on a medical school committee on Diversity and Professional Development. Throughout her career, she has been a mentor and sponsor to women and men in medical physics. In 2010, she was a founding member of the AAPM's Women's Professional Subcommittee.^1^ She spoke on gender equity in Medical Physics at Argonne National Laboratory in 2017. She is active in clinic service, leadership, research, and teaching.

Dr. Mary K. Martel is Professor and Chair of the Department of Radiation Physics in the Division of Radiation Oncology at M.D. Anderson Cancer Center. She has held top leadership roles as President and Chairwoman of the AAPM Board, and chair of Science Council for ASTRO. With Dr. Jean Moran, she organized a panel at the 2009 AAPM annual meeting “Professional Development for Women in the AAPM”. The Women's Professional Subcommittee of the AAPM was formed shortly after, and an annual Women Physicists Luncheon at the AAPM meeting is a successful forum for networking. One of Dr. Martel's passions is mentorship and she is proud to be a mentor to many successful male and female faculties.

## 2 CALL FOR ACTION: DEFINING ROLES FOR MEN AND WOMEN IN MEDICAL PHYSICS TO PROMOTE GENDER EQUITY

### 2.A Julianne Pollard‐Larkin, PhD

The news has been full of reports from brave women and one notably wrote the recent ASCO Post entitled “An Invitation to Be Quiet No Longer”.^3^ Dr. Stephanie Graff's article was a proverbial “call‐to‐arms” for women in Medicine. She stoically has urged all of us to start having the difficult conversations to give voice to the voiceless and do the work to make sexual harassment and gender discrimination a thing of the past in Medicine. On behalf of the rapidly growing numbers of young women trainees within Medical Physics and their more experienced women medical physicist role models, I and my co‐authors would like to tell her resoundingly, mission accepted!

Previously, I have written about how incredibly gender equitable Medical Physics is becoming and for this reason, I think we are the one medical STEM specialty that can take on Dr. Graff's challenge the best. With nearly 40% of all of our new trainees being women, we have created a broad base of support for all women trainees and a critical mass capable of inoculating themselves from feeling isolated and exposed to potential harassers.^4^ Across our field, we have about 75% of medical physicists being male, so although our training institutions may be more gender balanced, the field as a whole will take time to reflect the diversity and inclusion we see in our training programs.^2^ We need to create the gender‐bias training that will be effective in educating all of us, from trainees to professionals alike in best practices to prevent harassment based on gender and other traits (i.e. Ethnicity, sexual orientation, religion, age, etc.).

The good thing is that studies have already shown what types of training programs help end gender discrimination and harassment. Traditional gender‐bias training programs simply teach trainees what gender‐based discrimination and harassment is and can even have negative repercussions since they typically frame women in the workplace as victims and the men as powerful.^5^ Reinforcing gender stereotypes, albeit unintentionally, does not help end gender discrimination or sexual harassment, it simply causes men to bristle at the idea of being labeled abusers and women to feel disconnected from the “victim” title.^5^

What does work to help equip both men and women to prevent sexual harassment and discrimination is by following practices that treat women and men equally.^2^ By paying women equitable salaries and promoting women to leadership positions as warranted by their accomplishments can help show the entire workforce that we are all to be treated with the same amount of respect and deference.^5^ The only way to beat inequality is with equity. And this is something that AAPM strives to do by routinely surveying salaries of all of our members.

One of the best training programs found in some current studies involves “empowering the bystander”.^5^ The military, college campuses, and non‐profit organizations have used this technique to train observers of harassment on how to de‐escalate the situation, disrupt it, and support the victim.^5^ This newer tactic helps to prevent passive observers and make equity and safety everyone's responsibility. Soldiers who received the bystander training were more likely to report that they took action to stop the abuse and the training caused college students in one study to have long‐lasting changes in their perceptions of sexual violence as a result.^5^, ^6^ If people are given a role to play other than victim or abuser, there is a higher propensity that they will take the training seriously and even put it into practice.

With tools like these in our arsenal, we can make Medical Physics more equitable for all. There is too much at stake if we don't make our increasingly diverse field more inclusive and safe for all.

We have a huge supply of potential male allies if they are open to learning about the prevalence of sexual harassment and how gender equity can be a win for them and women alike. For far too long, gender equality has been synonymous with women's empowerment, allowing men to inadvertently not recognize the advantages they will enjoy should they help us make STEM more gender equitable. Gender equity can benefit more than just STEM, it can benefit the global market, studies have estimated that we could potentially add up $12 trillion dollars to the global economy if we reached gender equity.^7^

Gender equity is an issue for us all, but given men's key roles in the bulk of all leadership positions, we simply cannot achieve the goal of gender equity without their help. Men are the CEO's of 95% of Fortune 500 companies and they control 81% of congress.^8^ Men are essential to this task. Men must awaken to the reality that they are stakeholders in this and that they are gendered too. Men face gender‐based stereotypes which prevent some men from entering fields that are considered “feminine” such as nursing and teaching kindergarten. In Austria, it was reported that less than 2% of all kindergarten teachers were male.^9^ In fact, according to some conversations I have had, some men outside of our field of Medical Physics have even considered it to be “girly” or less rigorous than traditional Pure Physics, as if there were some masculinity scale of Physics with Quantum Physics on the top and Medical Physics on the bottom. This patriarchal framework is simply perceived biases gone awry. We need to see the underlying issue here that connecting negative connotations to femininity or womanhood hurts us all. We have nothing to gain by supporting gender stereotypes and biased attitudes.

Men need the most effort and support at this junction as we prepare to support this new influx of women medical physicist trainees. Most women scientists are connected to and are aware of mentor programs, women in STEM‐focused resources and support groups such as AAPM's Women's Professional Subcommittee. Men, however, are far less likely to know about gender inequity issues, unconscious bias training, and the benefits they could gain from being an ally to gender equity pursuits. We need to create programming for men and be available to listen to their concerns as they deal with the changing gender dynamics within the field.

Most importantly, we should take heed to the Council of the European Union's conclusions on men and gender equality: “in order to improve the status of women and promote gender equality, more attention should be paid to how men are involved in the achievement of gender equality, as well as to the positive impact of gender equality for men and for the well‐being of society as a whole”.^10^ We need to focus on our men and help make them feel empowered to assist us with making Medical Physics even more gender equitable.

## 3 GENDER EQUITY IN MEDICAL PHYSICS: A ROADMAP FOR CHANGE

### 3.A Kelly C. Paradis, PhD

Over the years, our field has made substantial positive strides toward achieving gender equity. For example, in reviewing the “about” page on http://www.aapm.org, you'll notice that two‐thirds of the 2018 executive committee, about 40% of the board of directors, and half of the presidential chain are women. One of the strategic goals of AAPM is now specifically to “promote diversity, inclusion, and equity in healthcare”. There are also the Diversity and Inclusion Subcommittee (chaired by Dr. Julianne Pollard‐Larkin), the Women's Professional Subcommittee (chaired by Dr. Laura Cervino), and a number of mentorship and fellowship programs aimed at enhancing diversity in our field. For the first time, both AAPM and ASTRO arranged for childcare at their respective 2018 annual meetings, enabling members to attend these meetings who might otherwise not have been able. Many individual institutions are also tackling gender equity issues directly, medical physicists are reaching out to the next generation of female scientists to offer resources and support, and I personally am incredibly fortunate to have had multiple amazing female role models and mentors throughout my career in the sciences.

So, have we solved the gender issue in our corner of STEM fields, or do we have more work to do? As a junior female medical physicist, I am frequently presented with the message that gender equity issues no longer exist for women in the sciences, or even in the general workforce. In fact, a recent Pew Research study^11^ reported that 45% of Americans think that “the obstacles that make it harder for women to get ahead are now largely gone” (56% of men agreed with this compared to 34% of women). Last year, a software engineer at Google distributed his anti‐diversity manifesto that claimed the gender gap in tech is simply due to biological differences (women's higher level of agreeableness and neuroticism, obviously).^12^ On October 1st of this year, CERN suspended a scientist for his statement at a conference on high energy physics and gender that physics was “invented and built by men”.^13^ Women would be welcome once they proved themselves (e.g. “got Nobels”). The following day Donna Strickland was awarded the Nobel Prize in physics for her work in ultra‐short optical pulses, and called out the CERN scientist's claims as “silly”.^13^

As reference above by Dr. Pollard‐Larkin, I read the recent ASCO post article by Dr. Stephanie L. Graff with a mix of dismay and anger regarding the experiences she has endured throughout her career. I applaud her courage in writing her article. The need for sharing these types of experiences is great, but so too is the associated danger — both personally and professionally. It's so easy to say, “I'd never put up with that!”, from the safety of one's computer screen. But in the moment, when one of these experiences happens to you, and you are paralyzed with shock and fear and questioning your own self‐worth, it is not so simple. This is why I react with such surprise (albeit primarily internally) when I hear the absolutely absurd assertion that somehow the sciences are immune to such conduct, (“*this has never happened to you, has it?*”).

But how do we know it's happening, besides personal anecdotes, and conference hostility? There are many quantitative metrics that illustrate the gender inequality in our field, including gender distribution,^14^ research funding,^15^ and salary.^16^ A clear wage gap between male and female medical physicists is evident in both the United States and Canada in the AAPM Professional Surveys (https://www.aapm.org/pubs/surveys.asp). In biomedicine, where women and men are entering at approximately the same rates, there still exists a staggering “leaky pipeline” on the way up to the top, and “elite” male faculty choose to hire fewer women compared to female faculty.^17^ A 2016 article in The Atlantic 18 highlighted the fact that though not everyone is aware of it, sexual harassment in the sciences remains a serious and pervasive issue. The National Academies of Science, Engineering, and Medicine recently released a study that found that more than “50 percent of women faculty and staff and 20–50 percent of women students encounter or experience sexually harassing conduct in academia” and that the rate at which this occurs is similar to that in other workplaces.^19^

While there is no easy solution to our gender equity dilemma, there is no question that gender‐diverse organizations perform better.^20^ It is vitally important that we come to a united front and understand that, even if we have not been personally affected, gender disparities and discrimination clearly exist in our field. These efforts are for the sake of our patients, our colleagues, our families and friends. We should endeavor to promote an open dialogue where all members of the field feel comfortable discussing their personal experiences and asking questions. The topic of gender equity is an area where quantitative data do not tell us the whole story, and therefore this open and honest discussion is crucially important. As an example, simply knowing the ratio of men to women entering the field does not tell us about the unique struggles that each group has encountered along the way (i.e., gender equality vs gender equity). Do women need and/or receive differing types of mentorship support than men? How have family commitments affected the career paths of both groups? What would senior medical physicists say to their younger counterparts about work/life integration and career success? Why does grant funding appear to differ between genders? And fundamentally, why do we disagree on whether gender equity is an issue in our field at all?

Moving forward, we should aim to create broad and diverse mentorship networks within our field, benefiting both junior, and senior medical physicists alike.^21^ Women need to help other women by avoiding social distancing (e.g., “I'm not like a typical woman, I don't…)^22^ and helping to create a culture where women support one another.^23^ We all need education regarding the unconscious biases that may inadvertently lead to discrimination in recruitment and conducting daily work. We should consider whether the timing of “early career awards” may disadvantage certain groups because of family commitments, and we should also continue to strive toward improving work‐life integration for everyone in the field (after all, men want to change diapers too).^24^ Thanks to many phenomenal leaders in our field there is positive momentum toward improvement, and I am excited to see what will happen in the coming years as we work toward gender equity together.

I've been finishing up my part of this article on a cold, cloudy, September afternoon in Michigan. Tucked away in the corner of my secondary computer monitor is a video feed of my 4‐month‐old daughter's daycare. It's a new experience for all of us. And all four babies are asleep at once right now (a daycare miracle!). This tiny little girl has exploded my whole world in a way I never could have predicted, and she has taken my views on gender equity to an entirely new plane. What I want for her is that she never hears the phrase “girls can do science too!” because *of course girls can do science*. But both her parents are physicists so maybe she'll want to do something completely different. And all four babies are awake again now. How fast things change.

## 4 CREATING SAFE SPACES IN MEDICAL PHYSICS AND THE AAPM

### 4.A Jean M. Moran, PhD and Mary K. Martel, PhD

A broad approach is needed toward gender equity which encompasses men and women in the AAPM. It is essential that we treat each other with compassion and respect and that we keep our goals in mind. We each bring a different lens to our work whether we have a single or blended emphasis on education such as focusing on creating the next generation leaders, on research such as innovating to bring new tools to launch advancements in patient care, on clinical care such as ensuring patients receive outstanding care in our respective specialties, or in government such as supporting regulatory and safety efforts to provide guidance to manufacturers in support of safety. We are collectively medical physicists in a field which thrives on team work. We feel privileged each day to be part of such a dynamic community.

The lack of gender equity and the presence of behaviors such as sexual harassment result in a spectrum of inequities that prevent individuals from bringing their best selves to perform our daily work. Working on the problem in only one way such as only men or only women will not get us to equity. Alongside fundamental medical physics books such as Khan and Johns and Cunningham, Jean's bookshelves include texts such as “Why So Slow?” by Virginia Valian^25^ which presents a stunning and what we have witnessed to be an accurate picture of how the same behaviors can be interpreted as positive or negative depending on if it's done by a woman or man (see the section on travel!); “Tempered Radicals” by Debra Meyerson on inspiring change in our work environments^26^; and “Ask for It” by Linda Babcock and Sara Laschever which proposes strategies for individuals to address gender inequities in their own lives.^27^ These books distill the findings of numerous studies and then present strategies for improvement. Practically, we see inequity time and again such as when women do not ask for what they need to do their work, when they are uncomfortable formulating their requests when negotiating, or when they hear their great idea being first ignored and then applauded when repeated as new by a male colleague.

Gender equity education for all gives us the opportunity to create safe spaces for both men and women. For individuals experiencing a challenging situation such as harassment, it's crucial that she or he has someone to talk to who can advise her or him on the situation. This could be someone in leadership or, human resources, a counselor, a trusted friend, or an independent coach. We have experienced the importance of having a trusted ear outside of a situation to strategize on how to address sexual harassment in our educational and clinical environments. We are collectively responsible for the creation and maintenance of these safe professional spaces.

Within the AAPM, we have observed how these senior women and men have treated each other with respect and how they are always looking for the next person to support. We look forward to further improvements within our specialty across our practice environments. Strategies for improvement can be found in the Consensus Study Report by the National Academies of Sciences, Engineering, and Medicine Report on Sexual Harassment.^19^ The AAPM has been taking significant steps toward equity and creating those safe, supportive spaces for our members to thrive. At this year's annual meeting, all attendees were made aware of our updated policy on harassment. Our Board of Directors recently approved an addition on Diversity, Equity, and Inclusion (DEI) in our Strategic Plan. We are excited to see the positive advances in DEI within the AAPM.

## NOTE

1 To learn more about the WPSC, see the newsletters at: https://www.aapm.org/pubs/WPSCNewsletter.asp.

## References

[1] Pollard‐Larkin J . https://www.astro.org/ASTRO/media/ASTRO/News%20and%20Publications/ASTROnews/PDFs/18Summer_ASTROnews.pdf; accessed 10/03/2018; 2018.

[2] Ozturk N , Armato SG 3rd , Giger ML , Serago CF , Ross LF . Ethics and professionalism in medical physics: a survey of AAPM members. Med Phys. 2013;40:047001. 23556930 10.1118/1.4797463 PMC3625237

[3] Graff L . An Invitation to Be Quiet No Longer. http://www.ascopost.com/issues/September-25-2018/an-invitation-to-be-quiet-no-longer/; accessed 10/05/2018.

[4] Dogan N . 2017 SDAMPP/CAMPEP Annual Residency Programs Report; http://www.sdampp.org/resources.php; accessed 08/29/2018.

[5] Miller C . Sexual Harassment Training Doesn't Work. But Some Things Do. https://www.nytimes.com/2017/12/11/upshot/sexual-harassment-workplace-prevention-effective.html; accessed 10/5/2018.

[6] Potter SJ , Moynihan MM . Bringing in the bystander in‐person prevention program to a US military installation: results from a pilot study. Mil Med. 2011;176:870–875. 21882775 10.7205/milmed-d-10-00483

[7] Roy K , Kimmel M , Johnson S . https://www.forbes.com/sites/ellevate/2018/05/09/the-importance-of-male-voices-in-the-gender-equity-narrative/#409448b029c1; Accessed 09/24/2018.

[8] Lui R . http://www.msnbc.com/msnbc/gender-equality-men; Accessed 09/24/2018.

[9] Anderson L . https://www.reuters.com/article/us-un-men-genderequality/gender-equality-whats-in-it-for-men-idUSKBN0M80A720150312; accessed 09/24/2018.

[10] Fronteddu C . https://timeforequality.org/news/the-role-of-men-in-gender-equality/; accessed 09/24/2018.

[11] Fingerhut H . In both parties, men and women differ over whether women still face obstacles to progress. http://www.pewresearch.org/fact-tank/2016/08/16/in-both-parties-men-and-women-differ-over-whether-women-still-face-obstacles-to-progress/, accessed 08/29/2018.

[12] Wakabayashi D . Google Fires Engineer Who Wrote Memo Questioning Women in Tech. https://www.nytimes.com/2017/08/07/business/google-women-engineer-fired-memo.html, accessed 08/29/2018.

[13] Castelvecchi D . ERN Suspends Italian Physicist Over Remarks Seen as Sexist. https://www.scientificamerican.com/article/cern-suspends-italian-physicist-over-remarks-seen-as-sexist/; accessed 10/05/2018.

[14] Tsapaki V , Rehani MM . Female medical physicists: the results of a survey carried out by the International Organization for Medical Physics. Phys Med. 2015;31:368–373. 25790723 10.1016/j.ejmp.2015.02.009

[15] Whelan B , Moros EG , Fahrig R , et al. Development and testing of a database of NIH research funding of AAPM members: a report from the AAPM Working Group for the Development of a Research Database (WGDRD). Med Phys. 2017;44:1590–1601. 28074545 10.1002/mp.12098 PMC9559539

[16] https://www.nsf.gov/statistics/2018/nsf18304/data/tab48.pdf; accessed 08/29/2018.

[17] Sheltzer JM , Smith JC . Elite male faculty in the life sciences employ fewer women. Proc Natl Acad Sci USA. 2014;111:10107–10112. 24982167 10.1073/pnas.1403334111 PMC4104900

[18] Williams JC , Massinger K . How Women Are Harassed Out of Science. https://www.theatlantic.com/science/archive/2016/07/how-women-are-harassed-out-of-science/492521/, accessed 08/29/2018.

[19] Johnson PA , Widnall SE , Benya FF . Sexual Harassment of Women: Climate, Culture, and Consequences in Academic Science, Engineering, and Medicine. Washington, D. C.: National Academies Press; 2018. 29894119

[20] Hunt V , Layton D , Prince S . Diversity Matters. https://www.mckinsey.com/~/media/mckinsey/business%20functions/organization/our%20insights/why%20diversity%20matters/diversity%20matters.ashx; 2015.

[21] DeCastro R , Sambuco D , Ubel PA , Stewart A , Jagsi R . Mentor networks in academic medicine: moving beyond a dyadic conception of mentoring for junior faculty researchers. Acad Med. 2013;88:488–496. 23425990 10.1097/ACM.0b013e318285d302 PMC3610810

[22] Rhoton LA . Distancing as a gendered barrier understanding women scientists’ gender practices. Gender Soc. 2011;25:696–716.

[23] https://500womenscientists.org/who-we-are/, accessed 08/29/2018.

[24] Kutcher A . Stop Gender Stereotyping: Provide Universally Accessible Changing Tables in All Your Stores. https://www.change.org/p/bethechange-provide-universally-accessible-changing-tables-in-all-your-stores.

[25] Valian V . Why so slow? The advancement of women. Cambridge, UK: MIT Press; 1999.

[26] Meyerson DE . The stanford organizational studies community: reflections of a tempered radical. Res Sociol Organ‐Res. 2010;28:365–372.

[27] Babcock L , Laschever S . Ask for it: How Women Can Use the Power of Negotiation to Get what They Really Want. New York, NY: Bantam Dell; 2008.

